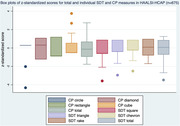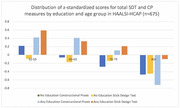# Measuring Visuospatial Ability in Low‐Literacy Older Adults: A Comparison of the Constructional Praxis and Stick Design Tests in Rural South Africa

**DOI:** 10.1002/alz.093097

**Published:** 2025-01-03

**Authors:** Darina T. Bassil, Meagan T. Farrell, Muqi Guo, Sarah Gao, Nomsa Mahlalehla, Adam M. Brickman, Jennifer J. Manly, Lisa F. Berkman

**Affiliations:** ^1^ Harvard T.H. Chan School of Public Health, Cambridge, MA USA; ^2^ IQVIA, New York, NY USA; ^3^ Harvard T. H. Chan School of Public Health, Cambridge, MA USA; ^4^ University of the Witwatersrand, Johannesburg South Africa; ^5^ Columbia University, New York, NY USA; ^6^ Department of Neurology, College of Physicians and Surgeons, Columbia University, New York, NY USA; ^7^ The Gertrude H. Sergievsky Center, College of Physicians and Surgeons, Columbia University, New York, NY USA; ^8^ Department of Neurology, Vagelos College of Physicians and Surgeons, Columbia University, and the New York Presbyterian Hospital, New York, NY USA; ^9^ Department of Neurology, Columbia University, New York, NY USA; ^10^ Department of Neurology, Columbia University Irving Medical Center, New York, NY USA; ^11^ Taub Institute for Research on Alzheimer’s Disease and the Aging Brain, Columbia University, New York, NY USA; ^12^ Taub Institute for Research on Alzheimer’s Disease and the Aging Brain, New York, NY USA; ^13^ Department of Neurology, Vagelos College of Physicians and Surgeons, Columbia University, New York Presbyterian Hospital, New York, NY USA

## Abstract

**Background:**

Dementia criteria require not only memory impairment, but additional impairment in at least one other cognitive domain, like visuospatial functioning. Cognitive measures assessing visuospatial function often involve drawing shapes. While these measures have proven reliable and valid in developed countries, their use in Low and Middle‐income Countries (LMICs) is challenging due to cultural differences and low literacy. Cultural and educational bias in visuospatial tests prevents comprehensive assessment of neuropsychological function, thus limiting dementia assessment in LMICs. Here, we compare the Stick Design Test (SDT) to Constructional Praxis (CP) for assessing visuospatial functioning among older South Africans adults.

**Method:**

We used data from Cognition and Dementia in the Health and Aging in Africa: A Longitudinal Study in South Africa (HAALSI‐HCAP), a population‐based nested cohort study in rural Agincourt, South Africa. In 2022, 675 participants, aged ≥ 50, were administered the immediate and delayed recall trials of the SDT and CP, as part of HAALSI‐HCAP cognitive battery. CP, completed on a tablet, involved copying four geometric shapes, while SDT required participants to reconstruct four configurations using matchsticks. Scoring for both CP (0‐11) and SDT (0‐12) was subsequently completed by trained researchers. We assessed acceptability and performance of both measures across participant demographics and investigated their correlation with performance on other visuospatial measures to ascertain criterion validity.

**Result:**

Participants were mostly women (63%), without education (58%) with mean age of 72.5±11. Refusals and missingness were more common for CP (40%) than SDT (13%). SDT had a more normal distribution compared to CP, overall and for individual figures. Worse performance was associated with higher age and lack of formal education for both measures. However, SDT demonstrated less extreme z‐standardized scores, particularly among those without education and over 70 years old. Participants were more likely to recall SDT configurations than CP figures after a delay. SDT showed weak but significant correlations (p<0.001) with CP(r = 0.14), Raven’s Matrices(r = 0.27), and Symbol Cancellation (r = 0.38).

**Conclusion:**

SDT outperformed CP in acceptability and demonstrated lower sensitivity to education. Convergent validity findings suggest that SDT may be a robust and culturally adaptable measure for assessing visuospatial functioning in older populations, especially in low‐resource settings.